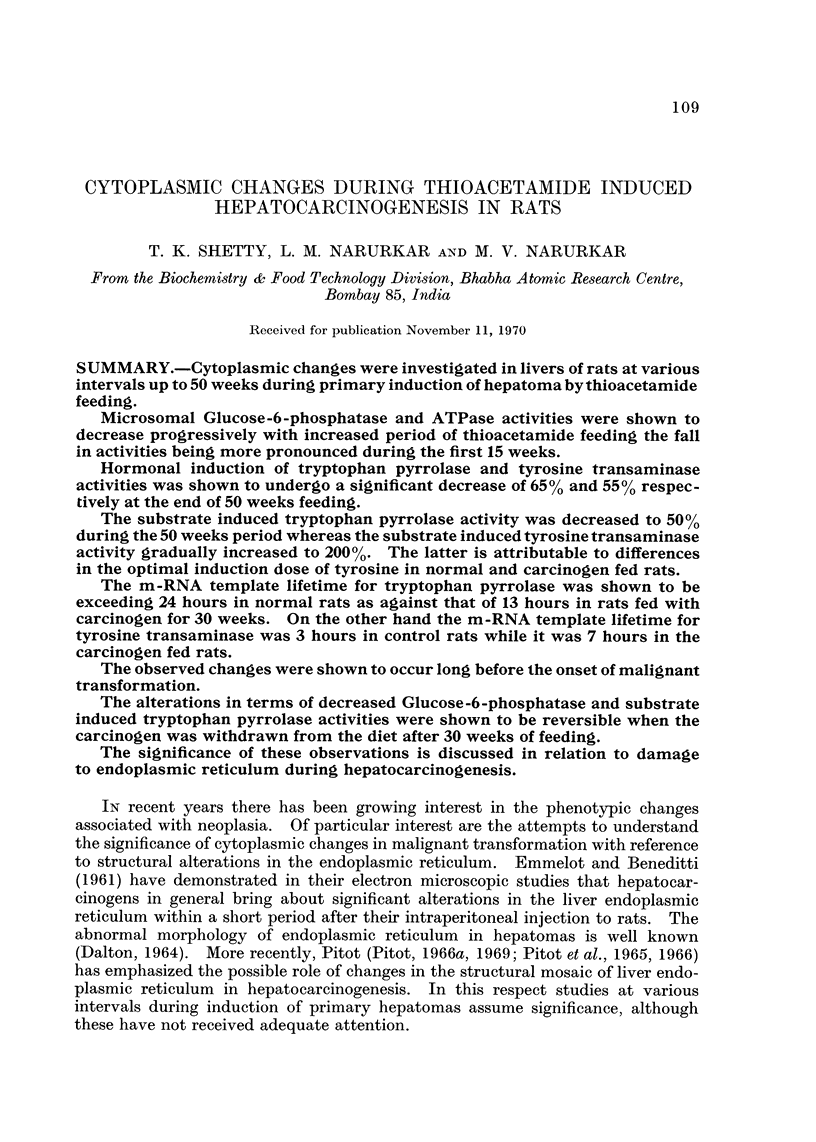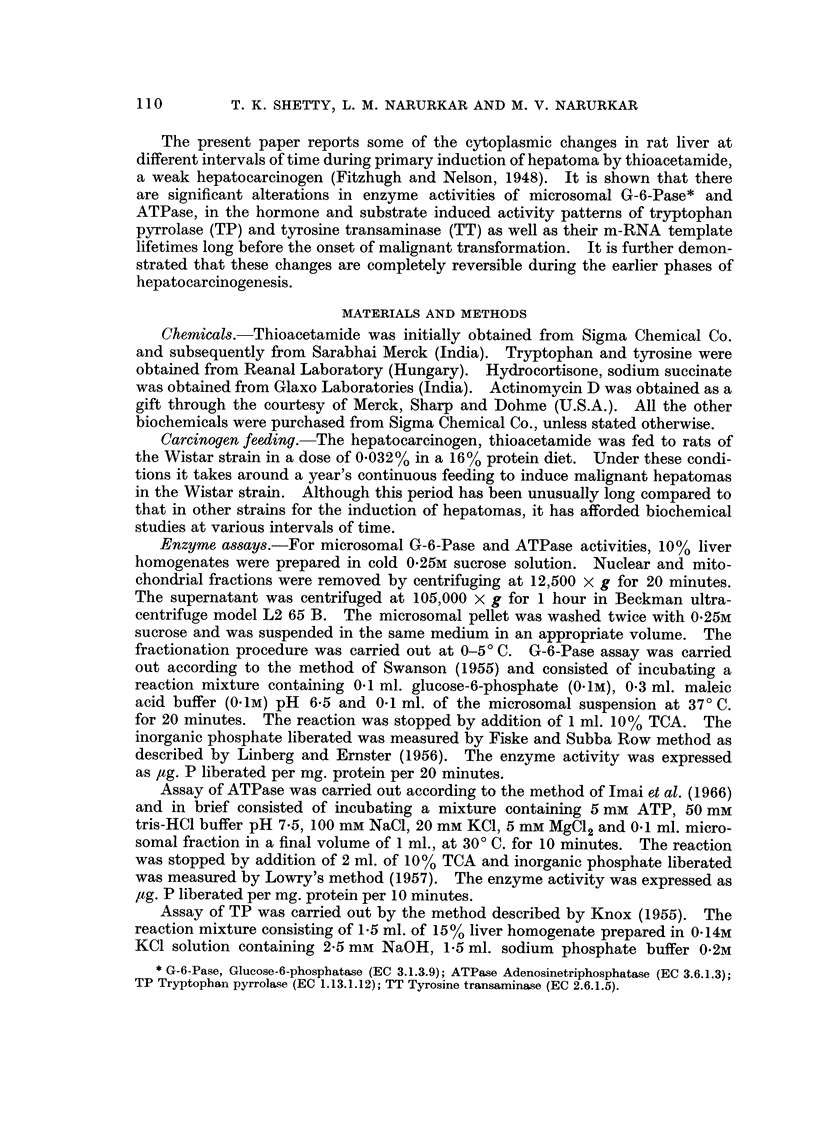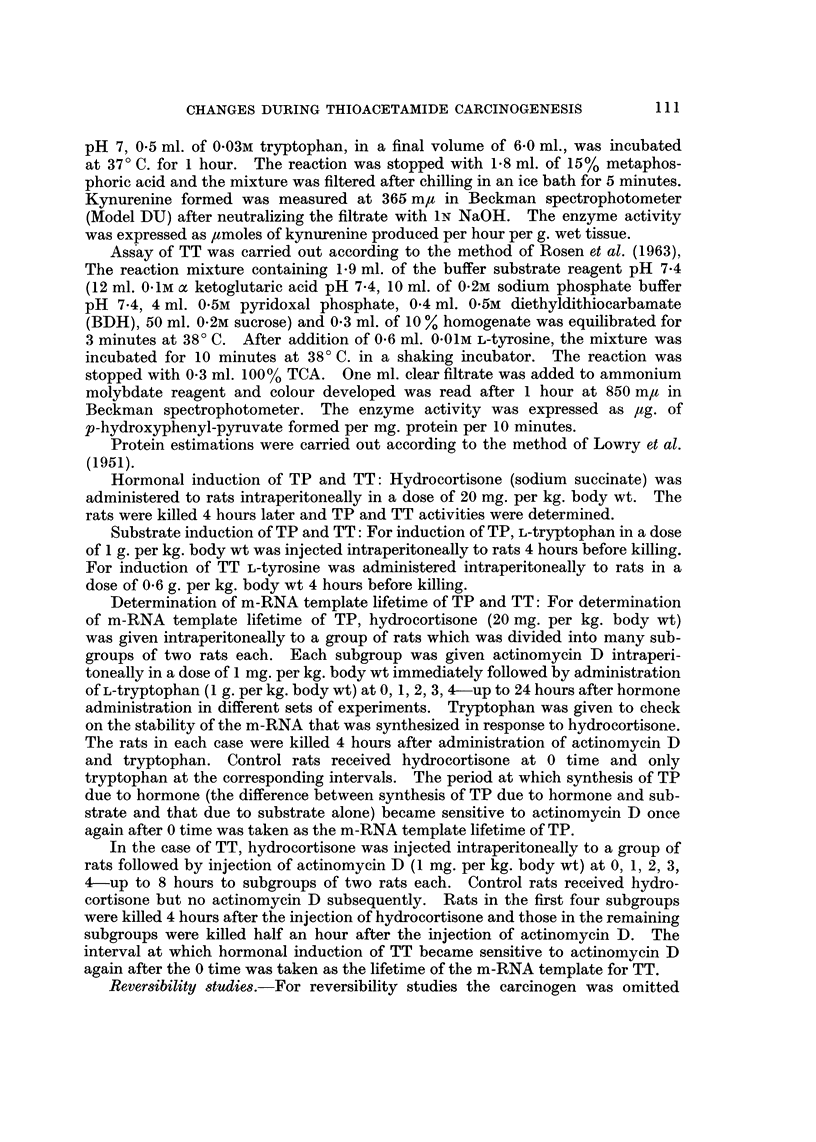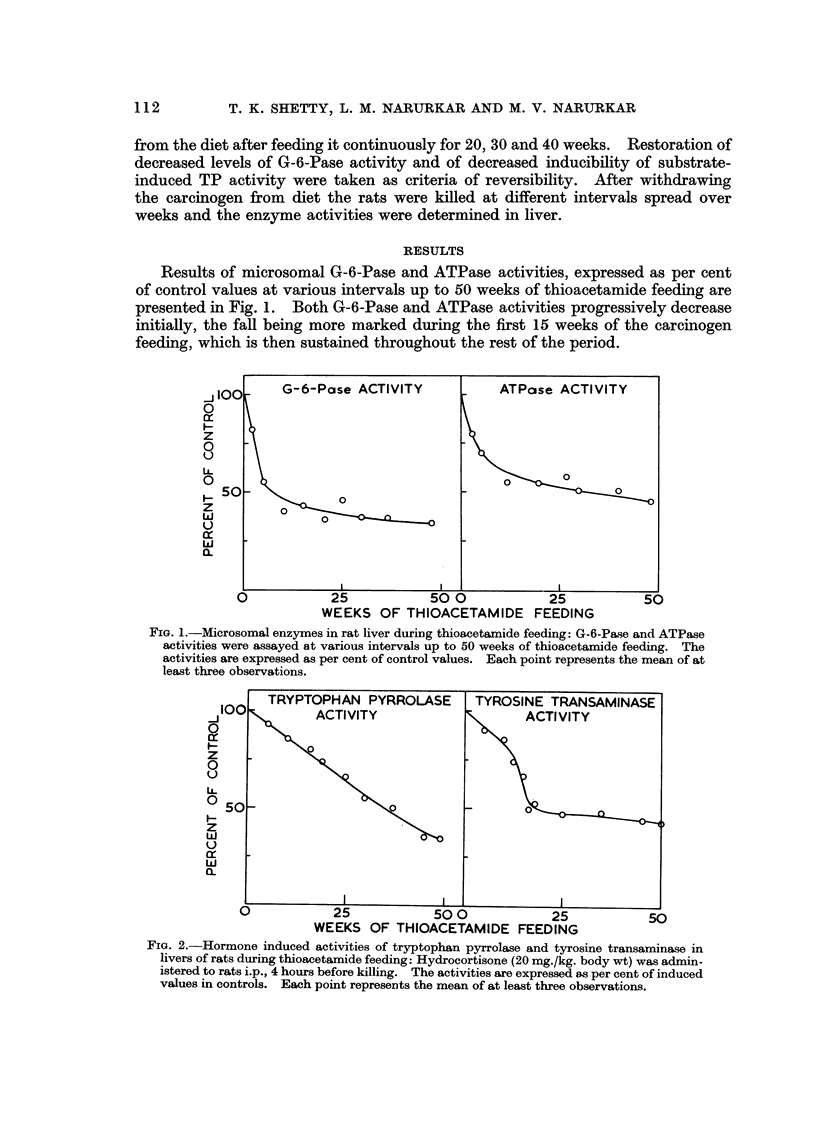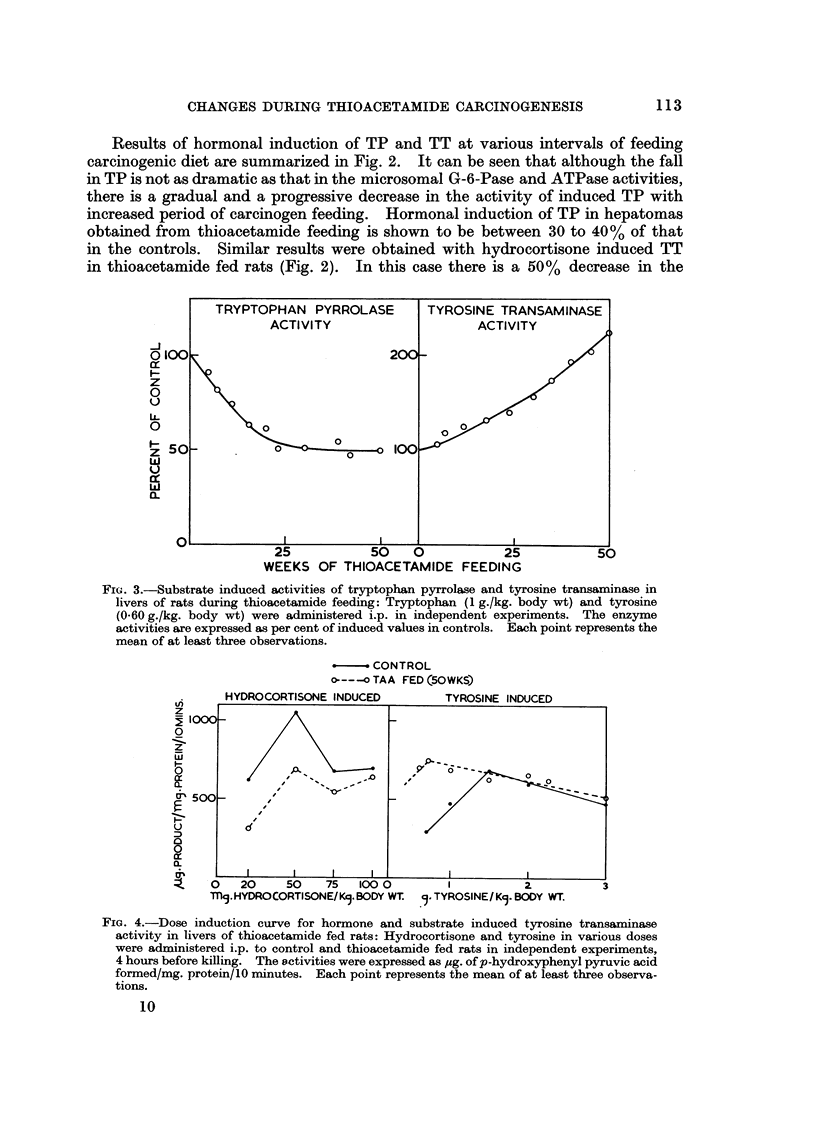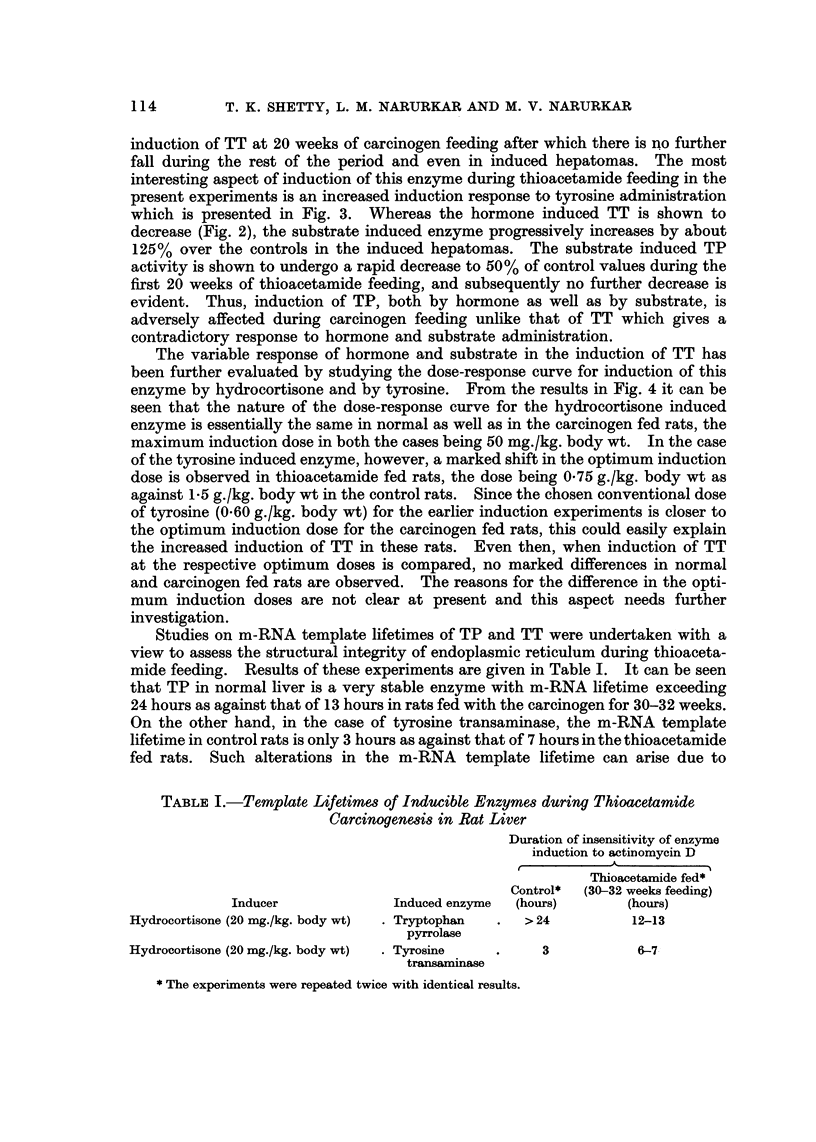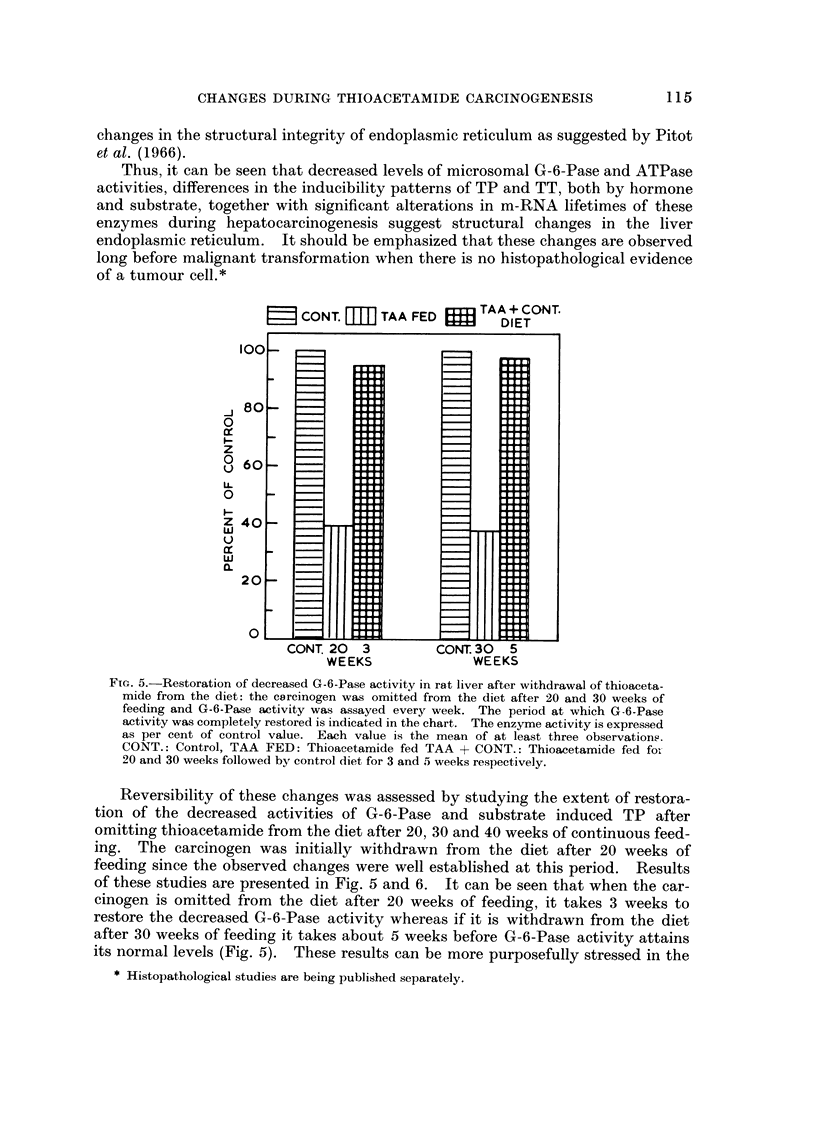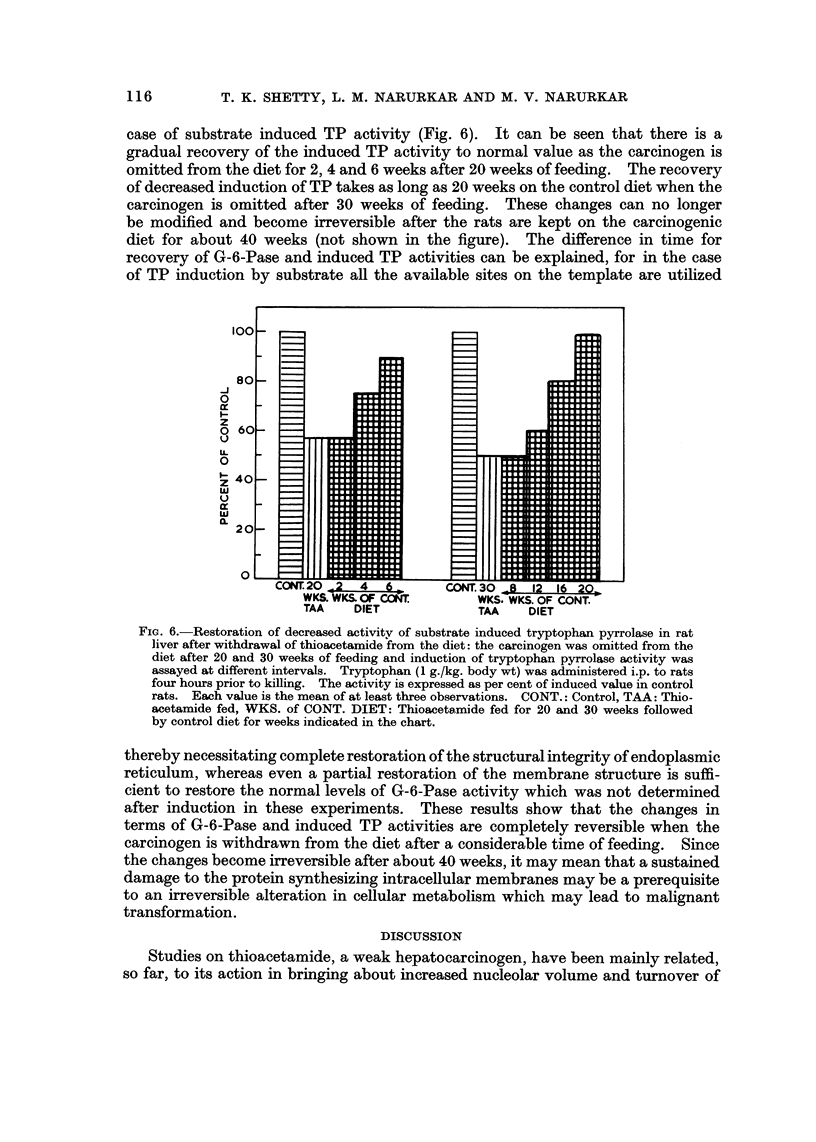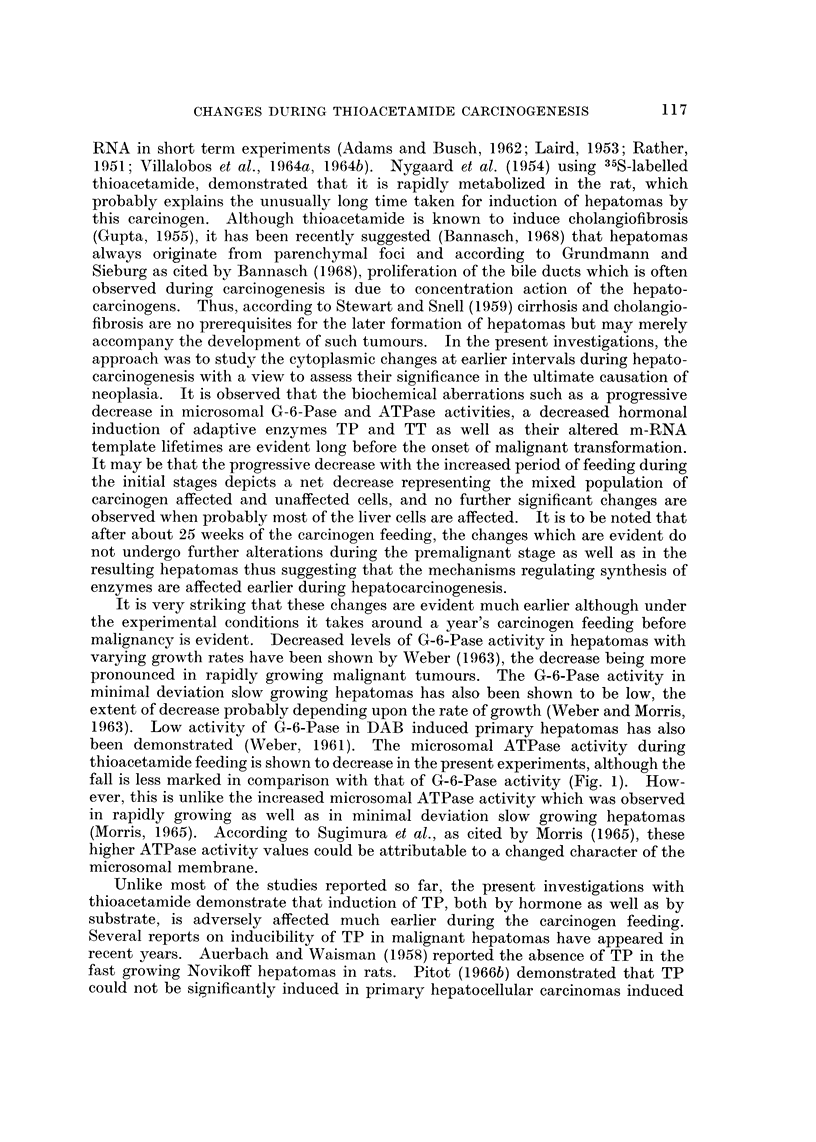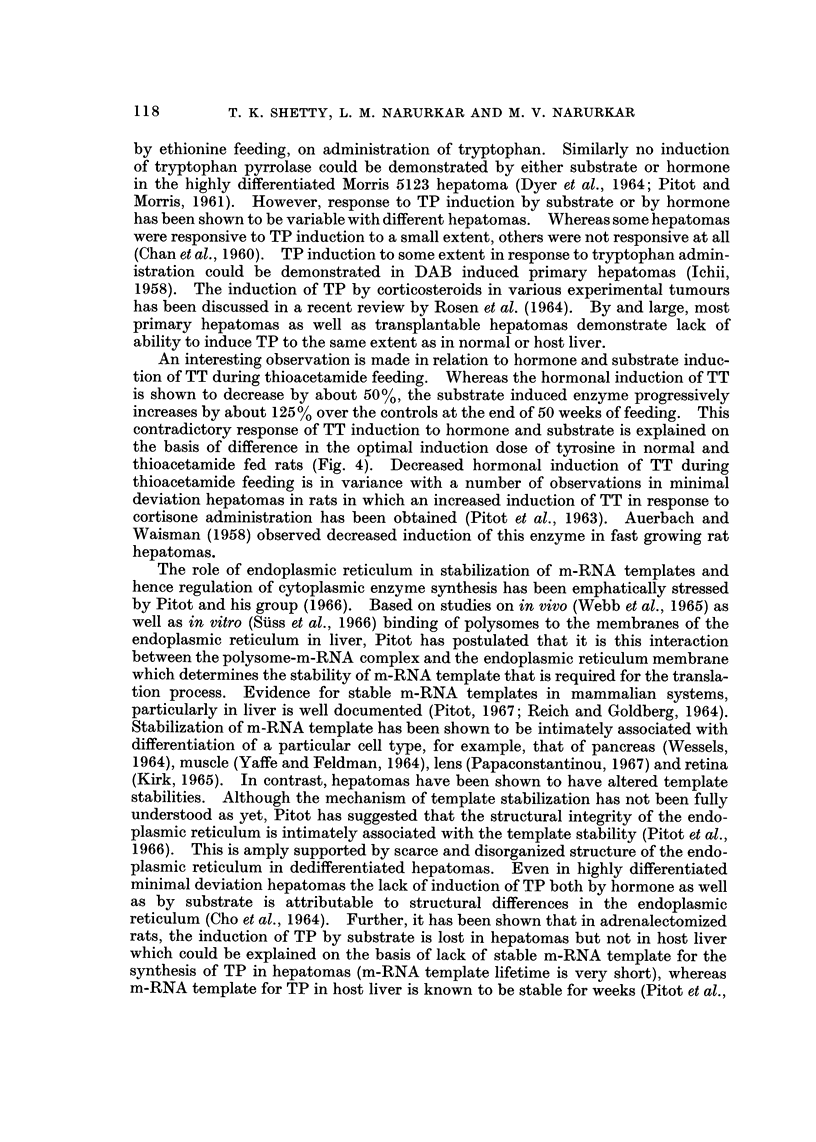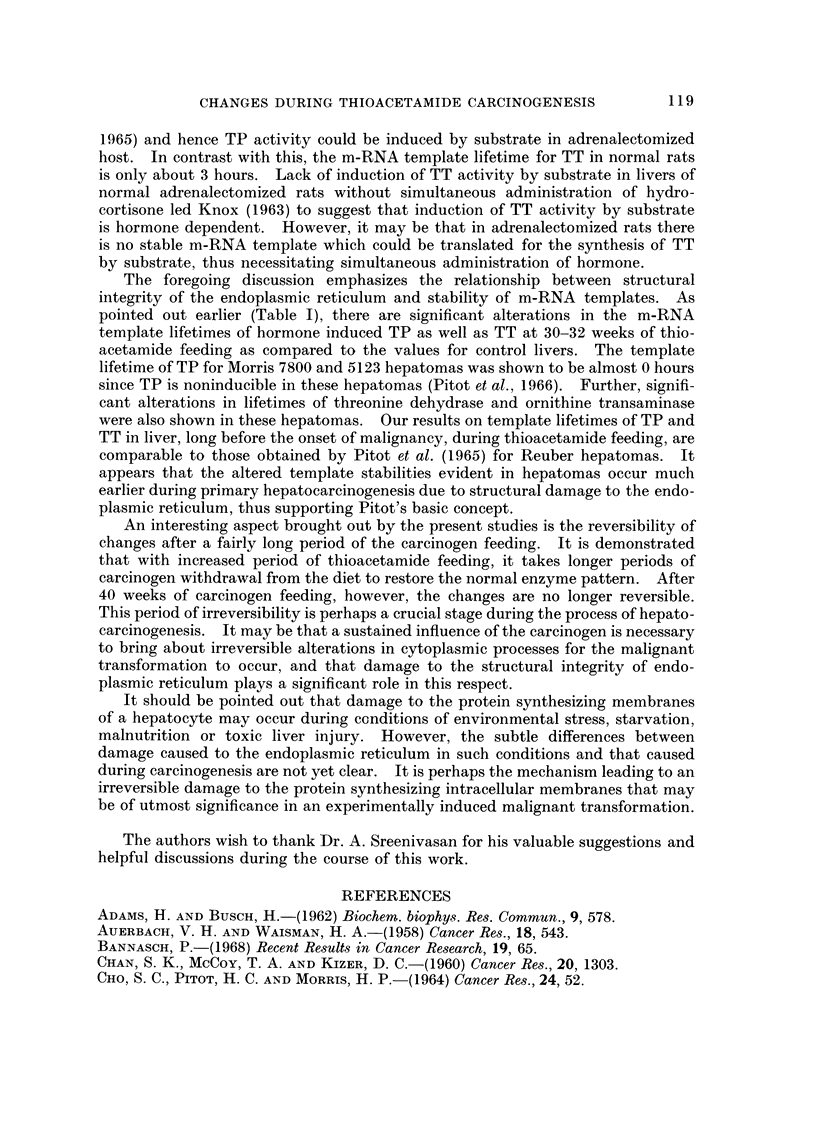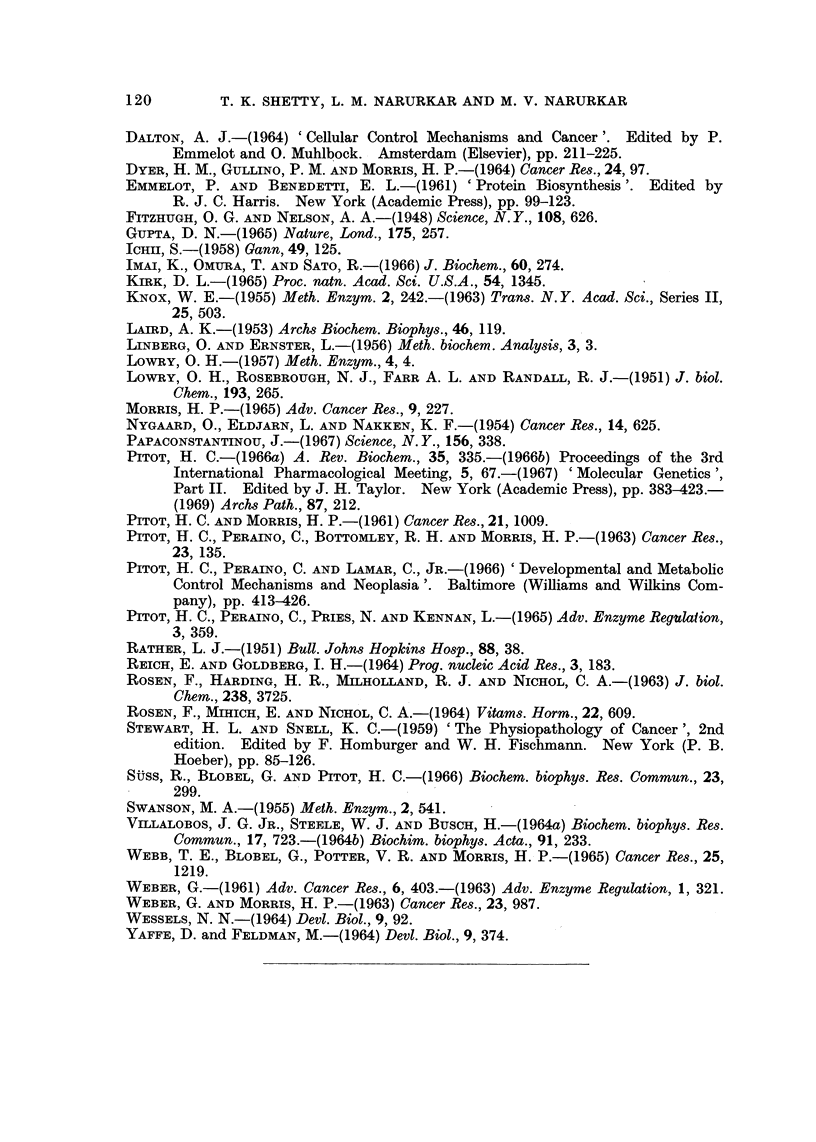# Cytoplasmic Changes During Thioacetamide Induced Hepatocarcinogenesis in Rats

**DOI:** 10.1038/bjc.1971.16

**Published:** 1971-03

**Authors:** T. K. Shetty, L. M. Narurkar, M. V. Narurkar

## Abstract

Cytoplasmic changes were investigated in livers of rats at various intervals up to 50 weeks during primary induction of hepatoma by thioacetamide feeding.

Microsomal Glucose-6-phosphatase and ATPase activities were shown to decrease progressively with increased period of thioacetamide feeding the fall in activities being more pronounced during the first 15 weeks.

Hormonal induction of tryptophan pyrrolase and tyrosine transaminase activities was shown to undergo a significant decrease of 65% and 55% respectively at the end of 50 weeks feeding.

The substrate induced tryptophan pyrrolase activity was decreased to 50% during the 50 weeks period whereas the substrate induced tyrosine transaminase activity gradually increased to 200%. The latter is attributable to differences in the optimal induction dose of tyrosine in normal and carcinogen fed rats.

The m-RNA template lifetime for tryptophan pyrrolase was shown to be exceeding 24 hours in normal rats as against that of 13 hours in rats fed with carcinogen for 30 weeks. On the other hand the m-RNA template lifetime for tyrosine transaminase was 3 hours in control rats while it was 7 hours in the carcinogen fed rats.

The observed changes were shown to occur long before the onset of malignant transformation.

The alterations in terms of decreased Glucose-6-phosphatase and substrate induced tryptophan pyrrolase activities were shown to be reversible when the carcinogen was withdrawn from the diet after 30 weeks of feeding.

The significance of these observations is discussed in relation to damage to endoplasmic reticulum during hepatocarcinogenesis.


### CYTOPLASMIC CHANGES DURING THIOACETAMIDE INDUCED HEPATOCARCINOGENESIS IN RATS

T. K. SHETTY, L. M. NARURKAR AND M. V. NARURKAR

*From the Biochemistry & Food Technology Division, Bhabha Atomic Research Centre, Bombay 85, India*

Received for publication November 11, 1970

**SUMMARY.—Cytoplasmic changes were investigated in livers of rats at various intervals up to 50 weeks during primary induction of hepatoma by thioacetamide feeding.**

**Microsomal Glucose-6-phosphatase and ATPase activities were shown to decrease progressively with increased period of thioacetamide feeding the fall in activities being more pronounced during the first 15 weeks.**

**Hormonal induction of tryptophan pyrrolase and tyrosine transaminase activities was shown to undergo a significant decrease of 65% and 55% respectively at the end of 50 weeks feeding.**

**The substrate induced tryptophan pyrrolase activity was decreased to 50% during the 50 weeks period whereas the substrate induced tyrosine transaminase activity gradually increased to 200%. The latter is attributable to differences in the optimal induction dose of tyrosine in normal and carcinogen fed rats.**

**The m-RNA template lifetime for tryptophan pyrrolase was shown to be exceeding 24 hours in normal rats as against that of 13 hours in rats fed with carcinogen for 30 weeks. On the other hand the m-RNA template lifetime for tyrosine transaminase was 3 hours in control rats while it was 7 hours in the carcinogen fed rats.**

**The observed changes were shown to occur long before the onset of malignant transformation.**

**The alterations in terms of decreased Glucose-6-phosphatase and substrate induced tryptophan pyrrolase activities were shown to be reversible when the carcinogen was withdrawn from the diet after 30 weeks of feeding.**

**The significance of these observations is discussed in relation to damage to endoplasmic reticulum during hepatocarcinogenesis.**

IN recent years there has been growing interest in the phenotypic changes associated with neoplasia. Of particular interest are the attempts to understand the significance of cytoplasmic changes in malignant transformation with reference to structural alterations in the endoplasmic reticulum. Emmelot and Beneditti (1961) have demonstrated in their electron microscopic studies that hepatocarcinogens in general bring about significant alterations in the liver endoplasmic reticulum within a short period after their intraperitoneal injection to rats. The abnormal morphology of endoplasmic reticulum in hepatomas is well known (Dalton, 1964). More recently, Pitot (Pitot, 1966*a*, 1969; Pitot *et al.*, 1965, 1966) has emphasized the possible role of changes in the structural mosaic of liver endoplasmic reticulum in hepatocarcinogenesis. In this respect studies at various intervals during induction of primary hepatomas assume significance, although these have not received adequate attention.

The present paper reports some of the cytoplasmic changes in rat liver at different intervals of time during primary induction of hepatoma by thioacetamide, a weak hepatocarcinogen (Fitzhugh and Nelson, 1948). It is shown that there are significant alterations in enzyme activities of microsomal G-6-Pase* and ATPase, in the hormone and substrate induced activity patterns of tryptophan pyrrolase (TP) and tyrosine transaminase (TT) as well as their m-RNA template lifetimes long before the onset of malignant transformation. It is further demonstrated that these changes are completely reversible during the earlier phases of hepatocarcinogenesis.

### MATERIALS AND METHODS

*Chemicals.*—Thioacetamide was initially obtained from Sigma Chemical Co. and subsequently from Sarabhai Merck (India). Tryptophan and tyrosine were obtained from Reanal Laboratory (Hungary). Hydrocortisone, sodium succinate was obtained from Glaxo Laboratories (India). Actinomycin D was obtained as a gift through the courtesy of Merck, Sharp and Dohme (U.S.A.). All the other biochemicals were purchased from Sigma Chemical Co., unless stated otherwise.

*Carcinogen feeding.*—The hepatocarcinogen, thioacetamide was fed to rats of the Wistar strain in a dose of 0·032% in a 16% protein diet. Under these conditions it takes around a year's continuous feeding to induce malignant hepatomas in the Wistar strain. Although this period has been unusually long compared to that in other strains for the induction of hepatomas, it has afforded biochemical studies at various intervals of time.

*Enzyme assays.*—For microsomal G-6-Pase and ATPase activities, 10% liver homogenates were prepared in cold 0·25M sucrose solution. Nuclear and mitochondrial fractions were removed by centrifuging at 12,500 × *g* for 20 minutes. The supernatant was centrifuged at 105,000 × *g* for 1 hour in Beckman ultracentrifuge model L2 65 B. The microsomal pellet was washed twice with 0·25M sucrose and was suspended in the same medium in an appropriate volume. The fractionation procedure was carried out at 0–5° C. G-6-Pase assay was carried out according to the method of Swanson (1955) and consisted of incubating a reaction mixture containing 0·1 ml. glucose-6-phosphate (0·1M), 0·3 ml. maleic acid buffer (0·1M) pH 6·5 and 0·1 ml. of the microsomal suspension at 37° C. for 20 minutes. The reaction was stopped by addition of 1 ml. 10% TCA. The inorganic phosphate liberated was measured by Fiske and Subba Row method as described by Linberg and Ernster (1956). The enzyme activity was expressed as μg. P liberated per mg. protein per 20 minutes.

Assay of ATPase was carried out according to the method of Imai *et al.* (1966) and in brief consisted of incubating a mixture containing 5 mM ATP, 50 mM tris-HCl buffer pH 7·5, 100 mM NaCl, 20 mM KCl, 5 mM $MgCl_2$ and 0·1 ml. microsomal fraction in a final volume of 1 ml., at 30° C. for 10 minutes. The reaction was stopped by addition of 2 ml. of 10% TCA and inorganic phosphate liberated was measured by Lowry's method (1957). The enzyme activity was expressed as μg. P liberated per mg. protein per 10 minutes.

Assay of TP was carried out by the method described by Knox (1955). The reaction mixture consisting of 1·5 ml. of 15% liver homogenate prepared in 0·14M KCl solution containing 2·5 mM NaOH, 1·5 ml. sodium phosphate buffer 0·2M

* G-6-Pase, Glucose-6-phosphatase (EC 3.1.3.9); ATPase Adenosinetriphosphatase (EC 3.6.1.3); TP Tryptophan pyrrolase (EC 1.13.1.12); TT Tyrosine transaminase (EC 2.6.1.5).

pH 7, 0·5 ml. of 0·03M tryptophan, in a final volume of 6·0 ml., was incubated at 37° C. for 1 hour. The reaction was stopped with 1·8 ml. of 15% metaphosphoric acid and the mixture was filtered after chilling in an ice bath for 5 minutes. Kynurenine formed was measured at 365 m$\mu$ in Beckman spectrophotometer (Model DU) after neutralizing the filtrate with 1N NaOH. The enzyme activity was expressed as $\mu$moles of kynurenine produced per hour per g. wet tissue.

Assay of TT was carried out according to the method of Rosen *et al.* (1963), The reaction mixture containing 1·9 ml. of the buffer substrate reagent pH 7·4 (12 ml. 0·1M $\alpha$ ketoglutaric acid pH 7·4, 10 ml. of 0·2M sodium phosphate buffer pH 7·4, 4 ml. 0·5M pyridoxal phosphate, 0·4 ml. 0·5M diethyldithiocarbamate (BDH), 50 ml. 0·2M sucrose) and 0·3 ml. of 10% homogenate was equilibrated for 3 minutes at 38° C. After addition of 0·6 ml. 0·01M L-tyrosine, the mixture was incubated for 10 minutes at 38° C. in a shaking incubator. The reaction was stopped with 0·3 ml. 100% TCA. One ml. clear filtrate was added to ammonium molybdate reagent and colour developed was read after 1 hour at 850 m$\mu$ in Beckman spectrophotometer. The enzyme activity was expressed as $\mu$g. of *p*-hydroxyphenyl-pyruvate formed per mg. protein per 10 minutes.

Protein estimations were carried out according to the method of Lowry *et al.* (1951).

Hormonal induction of TP and TT: Hydrocortisone (sodium succinate) was administered to rats intraperitoneally in a dose of 20 mg. per kg. body wt. The rats were killed 4 hours later and TP and TT activities were determined.

Substrate induction of TP and TT: For induction of TP, L-tryptophan in a dose of 1 g. per kg. body wt was injected intraperitoneally to rats 4 hours before killing. For induction of TT L-tyrosine was administered intraperitoneally to rats in a dose of 0·6 g. per kg. body wt 4 hours before killing.

Determination of m-RNA template lifetime of TP and TT: For determination of m-RNA template lifetime of TP, hydrocortisone (20 mg. per kg. body wt) was given intraperitoneally to a group of rats which was divided into many subgroups of two rats each. Each subgroup was given actinomycin D intraperitoneally in a dose of 1 mg. per kg. body wt immediately followed by administration of L-tryptophan (1 g. per kg. body wt) at 0, 1, 2, 3, 4—up to 24 hours after hormone administration in different sets of experiments. Tryptophan was given to check on the stability of the m-RNA that was synthesized in response to hydrocortisone. The rats in each case were killed 4 hours after administration of actinomycin D and tryptophan. Control rats received hydrocortisone at 0 time and only tryptophan at the corresponding intervals. The period at which synthesis of TP due to hormone (the difference between synthesis of TP due to hormone and substrate and that due to substrate alone) became sensitive to actinomycin D once again after 0 time was taken as the m-RNA template lifetime of TP.

In the case of TT, hydrocortisone was injected intraperitoneally to a group of rats followed by injection of actinomycin D (1 mg. per kg. body wt) at 0, 1, 2, 3, 4—up to 8 hours to subgroups of two rats each. Control rats received hydrocortisone but no actinomycin D subsequently. Rats in the first four subgroups were killed 4 hours after the injection of hydrocortisone and those in the remaining subgroups were killed half an hour after the injection of actinomycin D. The interval at which hormonal induction of TT became sensitive to actinomycin D again after the 0 time was taken as the lifetime of the m-RNA template for TT.

*Reversibility studies.*—For reversibility studies the carcinogen was omitted from the diet after feeding it continuously for 20, 30 and 40 weeks. Restoration of decreased levels of G-6-Pase activity and of decreased inducibility of substrate-induced TP activity were taken as criteria of reversibility. After withdrawing the carcinogen from diet the rats were killed at different intervals spread over weeks and the enzyme activities were determined in liver.

### RESULTS

Results of microsomal G-6-Pase and ATPase activities, expressed as per cent of control values at various intervals up to 50 weeks of thioacetamide feeding are presented in Fig. 1. Both G-6-Pase and ATPase activities progressively decrease initially, the fall being more marked during the first 15 weeks of the carcinogen feeding, which is then sustained throughout the rest of the period.

Fig. 1.—Microsomal enzymes in rat liver during thioacetamide feeding: G-6-Pase and ATPase activities were assayed at various intervals up to 50 weeks of thioacetamide feeding. The activities are expressed as per cent of control values. Each point represents the mean of at least three observations.

Fig. 2.—Hormone induced activities of tryptophan pyrrolase and tyrosine transaminase in livers of rats during thioacetamide feeding: Hydrocortisone (20 mg./kg. body wt) was administered to rats i.p., 4 hours before killing. The activities are expressed as per cent of induced values in controls. Each point represents the mean of at least three observations.

Results of hormonal induction of TP and TT at various intervals of feeding carcinogenic diet are summarized in Fig. 2. It can be seen that although the fall in TP is not as dramatic as that in the microsomal G-6-Pase and ATPase activities, there is a gradual and a progressive decrease in the activity of induced TP with increased period of carcinogen feeding. Hormonal induction of TP in hepatomas obtained from thioacetamide feeding is shown to be between 30 to 40% of that in the controls. Similar results were obtained with hydrocortisone induced TT in thioacetamide fed rats (Fig. 2). In this case there is a 50% decrease in the

Fig. 3.—Substrate induced activities of tryptophan pyrrolase and tyrosine transaminase in livers of rats during thioacetamide feeding: Tryptophan (1 g./kg. body wt) and tyrosine (0·60 g./kg. body wt) were administered i.p. in independent experiments. The enzyme activities are expressed as per cent of induced values in controls. Each point represents the mean of at least three observations.

Fig. 4.—Dose induction curve for hormone and substrate induced tyrosine transaminase activity in livers of thioacetamide fed rats: Hydrocortisone and tyrosine in various doses were administered i.p. to control and thioacetamide fed rats in independent experiments, 4 hours before killing. The activities were expressed as $\mu g.$ of $p$-hydroxyphenyl pyruvic acid formed/mg. protein/10 minutes. Each point represents the mean of at least three observations.

10

induction of TT at 20 weeks of carcinogen feeding after which there is no further fall during the rest of the period and even in induced hepatomas. The most interesting aspect of induction of this enzyme during thioacetamide feeding in the present experiments is an increased induction response to tyrosine administration which is presented in Fig. 3. Whereas the hormone induced TT is shown to decrease (Fig. 2), the substrate induced enzyme progressively increases by about 125% over the controls in the induced hepatomas. The substrate induced TP activity is shown to undergo a rapid decrease to 50% of control values during the first 20 weeks of thioacetamide feeding, and subsequently no further decrease is evident. Thus, induction of TP, both by hormone as well as by substrate, is adversely affected during carcinogen feeding unlike that of TT which gives a contradictory response to hormone and substrate administration.

The variable response of hormone and substrate in the induction of TT has been further evaluated by studying the dose-response curve for induction of this enzyme by hydrocortisone and by tyrosine. From the results in Fig. 4 it can be seen that the nature of the dose-response curve for the hydrocortisone induced enzyme is essentially the same in normal as well as in the carcinogen fed rats, the maximum induction dose in both the cases being 50 mg./kg. body wt. In the case of the tyrosine induced enzyme, however, a marked shift in the optimum induction dose is observed in thioacetamide fed rats, the dose being 0·75 g./kg. body wt as against 1·5 g./kg. body wt in the control rats. Since the chosen conventional dose of tyrosine (0·60 g./kg. body wt) for the earlier induction experiments is closer to the optimum induction dose for the carcinogen fed rats, this could easily explain the increased induction of TT in these rats. Even then, when induction of TT at the respective optimum doses is compared, no marked differences in normal and carcinogen fed rats are observed. The reasons for the difference in the optimum induction doses are not clear at present and this aspect needs further investigation.

Studies on m-RNA template lifetimes of TP and TT were undertaken with a view to assess the structural integrity of endoplasmic reticulum during thioacetamide feeding. Results of these experiments are given in Table I. It can be seen that TP in normal liver is a very stable enzyme with m-RNA lifetime exceeding 24 hours as against that of 13 hours in rats fed with the carcinogen for 30–32 weeks. On the other hand, in the case of tyrosine transaminase, the m-RNA template lifetime in control rats is only 3 hours as against that of 7 hours in the thioacetamide fed rats. Such alterations in the m-RNA template lifetime can arise due to

TABLE I.—*Template Lifetimes of Inducible Enzymes during Thioacetamide Carcinogenesis in Rat Liver*

| | | Duration of insensitivity of enzyme induction to actinomycin D | |
|---|---|---|---|
| Inducer | Induced enzyme | Control* (hours) | Thioacetamide fed* (30–32 weeks feeding) (hours) |
| Hydrocortisone (20 mg./kg. body wt) | Tryptophan pyrrolase | >24 | 12–13 |
| Hydrocortisone (20 mg./kg. body wt) | Tyrosine transaminase | 3 | 6–7 |

* The experiments were repeated twice with identical results.

changes in the structural integrity of endoplasmic reticulum as suggested by Pitot *et al.* (1966).

Thus, it can be seen that decreased levels of microsomal G-6-Pase and ATPase activities, differences in the inducibility patterns of TP and TT, both by hormone and substrate, together with significant alterations in m-RNA lifetimes of these enzymes during hepatocarcinogenesis suggest structural changes in the liver endoplasmic reticulum. It should be emphasized that these changes are observed long before malignant transformation when there is no histopathological evidence of a tumour cell.*

FIG. 5.—Restoration of decreased G-6-Pase activity in rat liver after withdrawal of thioacetamide from the diet: the carcinogen was omitted from the diet after 20 and 30 weeks of feeding and G-6-Pase activity was assayed every week. The period at which G-6-Pase activity was completely restored is indicated in the chart. The enzyme activity is expressed as per cent of control value. Each value is the mean of at least three observations. CONT.: Control, TAA FED: Thioacetamide fed TAA + CONT.: Thioacetamide fed for 20 and 30 weeks followed by control diet for 3 and 5 weeks respectively.

Reversibility of these changes was assessed by studying the extent of restoration of the decreased activities of G-6-Pase and substrate induced TP after omitting thioacetamide from the diet after 20, 30 and 40 weeks of continuous feeding. The carcinogen was initially withdrawn from the diet after 20 weeks of feeding since the observed changes were well established at this period. Results of these studies are presented in Fig. 5 and 6. It can be seen that when the carcinogen is omitted from the diet after 20 weeks of feeding, it takes 3 weeks to restore the decreased G-6-Pase activity whereas if it is withdrawn from the diet after 30 weeks of feeding it takes about 5 weeks before G-6-Pase activity attains its normal levels (Fig. 5). These results can be more purposefully stressed in the

* Histopathological studies are being published separately.

case of substrate induced TP activity (Fig. 6). It can be seen that there is a gradual recovery of the induced TP activity to normal value as the carcinogen is omitted from the diet for 2, 4 and 6 weeks after 20 weeks of feeding. The recovery of decreased induction of TP takes as long as 20 weeks on the control diet when the carcinogen is omitted after 30 weeks of feeding. These changes can no longer be modified and become irreversible after the rats are kept on the carcinogenic diet for about 40 weeks (not shown in the figure). The difference in time for recovery of G-6-Pase and induced TP activities can be explained, for in the case of TP induction by substrate all the available sites on the template are utilized

FIG. 6.—Restoration of decreased activity of substrate induced tryptophan pyrrolase in rat liver after withdrawal of thioacetamide from the diet: the carcinogen was omitted from the diet after 20 and 30 weeks of feeding and induction of tryptophan pyrrolase activity was assayed at different intervals. Tryptophan (1 g./kg. body wt) was administered i.p. to rats four hours prior to killing. The activity is expressed as per cent of induced value in control rats. Each value is the mean of at least three observations. CONT.: Control, TAA: Thioacetamide fed, WKS. of CONT. DIET: Thioacetamide fed for 20 and 30 weeks followed by control diet for weeks indicated in the chart.

thereby necessitating complete restoration of the structural integrity of endoplasmic reticulum, whereas even a partial restoration of the membrane structure is sufficient to restore the normal levels of G-6-Pase activity which was not determined after induction in these experiments. These results show that the changes in terms of G-6-Pase and induced TP activities are completely reversible when the carcinogen is withdrawn from the diet after a considerable time of feeding. Since the changes become irreversible after about 40 weeks, it may mean that a sustained damage to the protein synthesizing intracellular membranes may be a prerequisite to an irreversible alteration in cellular metabolism which may lead to malignant transformation.

### DISCUSSION

Studies on thioacetamide, a weak hepatocarcinogen, have been mainly related, so far, to its action in bringing about increased nucleolar volume and turnover of

RNA in short term experiments (Adams and Busch, 1962; Laird, 1953; Rather, 1951; Villalobos *et al.*, 1964*a*, 1964*b*). Nygaard *et al.* (1954) using $^{35}$S-labelled thioacetamide, demonstrated that it is rapidly metabolized in the rat, which probably explains the unusually long time taken for induction of hepatomas by this carcinogen. Although thioacetamide is known to induce cholangiofibrosis (Gupta, 1955), it has been recently suggested (Bannasch, 1968) that hepatomas always originate from parenchymal foci and according to Grundmann and Sieburg as cited by Bannasch (1968), proliferation of the bile ducts which is often observed during carcinogenesis is due to concentration action of the hepatocarcinogens. Thus, according to Stewart and Snell (1959) cirrhosis and cholangiofibrosis are no prerequisites for the later formation of hepatomas but may merely accompany the development of such tumours. In the present investigations, the approach was to study the cytoplasmic changes at earlier intervals during hepatocarcinogenesis with a view to assess their significance in the ultimate causation of neoplasia. It is observed that the biochemical aberrations such as a progressive decrease in microsomal G-6-Pase and ATPase activities, a decreased hormonal induction of adaptive enzymes TP and TT as well as their altered m-RNA template lifetimes are evident long before the onset of malignant transformation. It may be that the progressive decrease with the increased period of feeding during the initial stages depicts a net decrease representing the mixed population of carcinogen affected and unaffected cells, and no further significant changes are observed when probably most of the liver cells are affected. It is to be noted that after about 25 weeks of the carcinogen feeding, the changes which are evident do not undergo further alterations during the premalignant stage as well as in the resulting hepatomas thus suggesting that the mechanisms regulating synthesis of enzymes are affected earlier during hepatocarcinogenesis.

It is very striking that these changes are evident much earlier although under the experimental conditions it takes around a year's carcinogen feeding before malignancy is evident. Decreased levels of G-6-Pase activity in hepatomas with varying growth rates have been shown by Weber (1963), the decrease being more pronounced in rapidly growing malignant tumours. The G-6-Pase activity in minimal deviation slow growing hepatomas has also been shown to be low, the extent of decrease probably depending upon the rate of growth (Weber and Morris, 1963). Low activity of G-6-Pase in DAB induced primary hepatomas has also been demonstrated (Weber, 1961). The microsomal ATPase activity during thioacetamide feeding is shown to decrease in the present experiments, although the fall is less marked in comparison with that of G-6-Pase activity (Fig. 1). However, this is unlike the increased microsomal ATPase activity which was observed in rapidly growing as well as in minimal deviation slow growing hepatomas (Morris, 1965). According to Sugimura *et al.*, as cited by Morris (1965), these higher ATPase activity values could be attributable to a changed character of the microsomal membrane.

Unlike most of the studies reported so far, the present investigations with thioacetamide demonstrate that induction of TP, both by hormone as well as by substrate, is adversely affected much earlier during the carcinogen feeding. Several reports on inducibility of TP in malignant hepatomas have appeared in recent years. Auerbach and Waisman (1958) reported the absence of TP in the fast growing Novikoff hepatomas in rats. Pitot (1966*b*) demonstrated that TP could not be significantly induced in primary hepatocellular carcinomas induced by ethionine feeding, on administration of tryptophan. Similarly no induction of tryptophan pyrrolase could be demonstrated by either substrate or hormone in the highly differentiated Morris 5123 hepatoma (Dyer *et al.*, 1964; Pitot and Morris, 1961). However, response to TP induction by substrate or by hormone has been shown to be variable with different hepatomas. Whereas some hepatomas were responsive to TP induction to a small extent, others were not responsive at all (Chan *et al.*, 1960). TP induction to some extent in response to tryptophan administration could be demonstrated in DAB induced primary hepatomas (Ichii, 1958). The induction of TP by corticosteroids in various experimental tumours has been discussed in a recent review by Rosen *et al.* (1964). By and large, most primary hepatomas as well as transplantable hepatomas demonstrate lack of ability to induce TP to the same extent as in normal or host liver.

An interesting observation is made in relation to hormone and substrate induction of TT during thioacetamide feeding. Whereas the hormonal induction of TT is shown to decrease by about 50%, the substrate induced enzyme progressively increases by about 125% over the controls at the end of 50 weeks of feeding. This contradictory response of TT induction to hormone and substrate is explained on the basis of difference in the optimal induction dose of tyrosine in normal and thioacetamide fed rats (Fig. 4). Decreased hormonal induction of TT during thioacetamide feeding is in variance with a number of observations in minimal deviation hepatomas in rats in which an increased induction of TT in response to cortisone administration has been obtained (Pitot *et al.*, 1963). Auerbach and Waisman (1958) observed decreased induction of this enzyme in fast growing rat hepatomas.

The role of endoplasmic reticulum in stabilization of m-RNA templates and hence regulation of cytoplasmic enzyme synthesis has been emphatically stressed by Pitot and his group (1966). Based on studies on *in vivo* (Webb *et al.*, 1965) as well as *in vitro* (Süss *et al.*, 1966) binding of polysomes to the membranes of the endoplasmic reticulum in liver, Pitot has postulated that it is this interaction between the polysome-m-RNA complex and the endoplasmic reticulum membrane which determines the stability of m-RNA template that is required for the translation process. Evidence for stable m-RNA templates in mammalian systems, particularly in liver is well documented (Pitot, 1967; Reich and Goldberg, 1964). Stabilization of m-RNA template has been shown to be intimately associated with differentiation of a particular cell type, for example, that of pancreas (Wessels, 1964), muscle (Yaffe and Feldman, 1964), lens (Papaconstantinou, 1967) and retina (Kirk, 1965). In contrast, hepatomas have been shown to have altered template stabilities. Although the mechanism of template stabilization has not been fully understood as yet, Pitot has suggested that the structural integrity of the endoplasmic reticulum is intimately associated with the template stability (Pitot *et al.*, 1966). This is amply supported by scarce and disorganized structure of the endoplasmic reticulum in dedifferentiated hepatomas. Even in highly differentiated minimal deviation hepatomas the lack of induction of TP both by hormone as well as by substrate is attributable to structural differences in the endoplasmic reticulum (Cho *et al.*, 1964). Further, it has been shown that in adrenalectomized rats, the induction of TP by substrate is lost in hepatomas but not in host liver which could be explained on the basis of lack of stable m-RNA template for the synthesis of TP in hepatomas (m-RNA template lifetime is very short), whereas m-RNA template for TP in host liver is known to be stable for weeks (Pitot *et al.*,

1965) and hence TP activity could be induced by substrate in adrenalectomized host. In contrast with this, the m-RNA template lifetime for TT in normal rats is only about 3 hours. Lack of induction of TT activity by substrate in livers of normal adrenalectomized rats without simultaneous administration of hydrocortisone led Knox (1963) to suggest that induction of TT activity by substrate is hormone dependent. However, it may be that in adrenalectomized rats there is no stable m-RNA template which could be translated for the synthesis of TT by substrate, thus necessitating simultaneous administration of hormone.

The foregoing discussion emphasizes the relationship between structural integrity of the endoplasmic reticulum and stability of m-RNA templates. As pointed out earlier (Table I), there are significant alterations in the m-RNA template lifetimes of hormone induced TP as well as TT at 30–32 weeks of thioacetamide feeding as compared to the values for control livers. The template lifetime of TP for Morris 7800 and 5123 hepatomas was shown to be almost 0 hours since TP is noninducible in these hepatomas (Pitot *et al.*, 1966). Further, significant alterations in lifetimes of threonine dehydrase and ornithine transaminase were also shown in these hepatomas. Our results on template lifetimes of TP and TT in liver, long before the onset of malignancy, during thioacetamide feeding, are comparable to those obtained by Pitot *et al.* (1965) for Reuber hepatomas. It appears that the altered template stabilities evident in hepatomas occur much earlier during primary hepatocarcinogenesis due to structural damage to the endoplasmic reticulum, thus supporting Pitot's basic concept.

An interesting aspect brought out by the present studies is the reversibility of changes after a fairly long period of the carcinogen feeding. It is demonstrated that with increased period of thioacetamide feeding, it takes longer periods of carcinogen withdrawal from the diet to restore the normal enzyme pattern. After 40 weeks of carcinogen feeding, however, the changes are no longer reversible. This period of irreversibility is perhaps a crucial stage during the process of hepatocarcinogenesis. It may be that a sustained influence of the carcinogen is necessary to bring about irreversible alterations in cytoplasmic processes for the malignant transformation to occur, and that damage to the structural integrity of endoplasmic reticulum plays a significant role in this respect.

It should be pointed out that damage to the protein synthesizing membranes of a hepatocyte may occur during conditions of environmental stress, starvation, malnutrition or toxic liver injury. However, the subtle differences between damage caused to the endoplasmic reticulum in such conditions and that caused during carcinogenesis are not yet clear. It is perhaps the mechanism leading to an irreversible damage to the protein synthesizing intracellular membranes that may be of utmost significance in an experimentally induced malignant transformation.

The authors wish to thank Dr. A. Sreenivasan for his valuable suggestions and helpful discussions during the course of this work.

### REFERENCES

Adams, H. and Busch, H.—(1962) *Biochem. biophys. Res. Commun.*, **9**, 578.
Auerbach, V. H. and Waisman, H. A.—(1958) *Cancer Res.*, **18**, 543.
Bannasch, P.—(1968) *Recent Results in Cancer Research*, **19**, 65.
Chan, S. K., McCoy, T. A. and Kizer, D. C.—(1960) *Cancer Res.*, **20**, 1303.
Cho, S. C., Pitot, H. C. and Morris, H. P.—(1964) *Cancer Res.*, **24**, 52.

DALTON, A. J.—(1964) 'Cellular Control Mechanisms and Cancer'. Edited by P. Emmelot and O. Muhlbock. Amsterdam (Elsevier), pp. 211–225.
DYER, H. M., GULLINO, P. M. AND MORRIS, H. P.—(1964) *Cancer Res.*, **24**, 97.
EMMELOT, P. AND BENEDETTI, E. L.—(1961) 'Protein Biosynthesis'. Edited by R. J. C. Harris. New York (Academic Press), pp. 99–123.
FITZHUGH, O. G. AND NELSON, A. A.—(1948) *Science, N.Y.*, **108**, 626.
GUPTA, D. N.—(1965) *Nature, Lond.*, **175**, 257.
ICHII, S.—(1958) *Gann*, **49**, 125.
IMAI, K., OMURA, T. AND SATO, R.—(1966) *J. Biochem.*, **60**, 274.
KIRK, D. L.—(1965) *Proc. natn. Acad. Sci. U.S.A.*, **54**, 1345.
KNOX, W. E.—(1955) *Meth. Enzym.* **2**, 242.—(1963) *Trans. N.Y. Acad. Sci.*, Series II, **25**, 503.
LAIRD, A. K.—(1953) *Archs Biochem. Biophys.*, **46**, 119.
LINBERG, O. AND ERNSTER, L.—(1956) *Meth. biochem. Analysis*, **3**, 3.
LOWRY, O. H.—(1957) *Meth. Enzym.*, **4**, 4.
LOWRY, O. H., ROSEBROUGH, N. J., FARR A. L. AND RANDALL, R. J.—(1951) *J. biol. Chem.*, **193**, 265.
MORRIS, H. P.—(1965) *Adv. Cancer Res.*, **9**, 227.
NYGAARD, O., ELDJARN, L. AND NAKKEN, K. F.—(1954) *Cancer Res.*, **14**, 625.
PAPACONSTANTINOU, J.—(1967) *Science, N.Y.*, **156**, 338.
PITOT, H. C.—(1966*a*) *A. Rev. Biochem.*, **35**, 335.—(1966*b*) Proceedings of the 3rd International Pharmacological Meeting, **5**, 67.—(1967) 'Molecular Genetics', Part II. Edited by J. H. Taylor. New York (Academic Press), pp. 383–423.—(1969) *Archs Path.*, **87**, 212.
PITOT, H. C. AND MORRIS, H. P.—(1961) *Cancer Res.*, **21**, 1009.
PITOT, H. C., PERAINO, C., BOTTOMLEY, R. H. AND MORRIS, H. P.—(1963) *Cancer Res.*, **23**, 135.
PITOT, H. C., PERAINO, C. AND LAMAR, C., JR.—(1966) 'Developmental and Metabolic Control Mechanisms and Neoplasia'. Baltimore (Williams and Wilkins Company), pp. 413–426.
PITOT, H. C., PERAINO, C., PRIES, N. AND KENNAN, L.—(1965) *Adv. Enzyme Regulation*, **3**, 359.
RATHER, L. J.—(1951) *Bull. Johns Hopkins Hosp.*, **88**, 38.
REICH, E. AND GOLDBERG, I. H.—(1964) *Prog. nucleic Acid Res.*, **3**, 183.
ROSEN, F., HARDING, H. R., MILHOLLAND, R. J. AND NICHOL, C. A.—(1963) *J. biol. Chem.*, **238**, 3725.
ROSEN, F., MIHICH, E. AND NICHOL, C. A.—(1964) *Vitams. Horm.*, **22**, 609.
STEWART, H. L. AND SNELL, K. C.—(1959) 'The Physiopathology of Cancer', 2nd edition. Edited by F. Homburger and W. H. Fischmann. New York (P. B. Hoeber), pp. 85–126.
SÜSS, R., BLOBEL, G. AND PITOT, H. C.—(1966) *Biochem. biophys. Res. Commun.*, **23**, 299.
SWANSON, M. A.—(1955) *Meth. Enzym.*, **2**, 541.
VILLALOBOS, J. G. JR., STEELE, W. J. AND BUSCH, H.—(1964*a*) *Biochem. biophys. Res. Commun.*, **17**, 723.—(1964*b*) *Biochim. biophys. Acta.*, **91**, 233.
WEBB, T. E., BLOBEL, G., POTTER, V. R. AND MORRIS, H. P.—(1965) *Cancer Res.*, **25**, 1219.
WEBER, G.—(1961) *Adv. Cancer Res.*, **6**, 403.—(1963) *Adv. Enzyme Regulation*, **1**, 321.
WEBER, G. AND MORRIS, H. P.—(1963) *Cancer Res.*, **23**, 987.
WESSELS, N. N.—(1964) *Devl. Biol.*, **9**, 92.
YAFFE, D. and FELDMAN, M.—(1964) *Devl. Biol.*, **9**, 374.